# Primo software as a tool for Monte Carlo simulations of intensity modulated radiotherapy: a feasibility study

**DOI:** 10.1186/s13014-018-1021-2

**Published:** 2018-05-15

**Authors:** Alessandro Esposito, Sofia Silva, Jorge Oliveira, Joana Lencart, João Santos

**Affiliations:** 1 Radiation Oncology Department, Princess Alexandra Hospital, Brisbane, Australia; 2 Medical Physics, Radiobiology and Radiation Protection Group, IPO Porto, Porto, Portugal; 3 Research Center (CI-IPOP), Portuguese Oncology Institute of Porto (IPO Porto), Porto, Portugal; 4 Medical Physics Department, Portuguese Oncology Institute of Porto (IPO Porto), Porto, Portugal; 5 Instituto de Ciências Biomédicas Abel Salazar (ICBAS), University of Porto, Porto, Portugal

**Keywords:** IMRT, Monte Carlo, Quality assurance, PRIMO

## Abstract

**Background:**

IMRT provides higher dose conformation to the target and dose sparing to surrounding tissues than 3DCRT. Monte Carlo method in Medical Physics is not a novelty to approach dosimetric problems. A new PENELOPE based code named PRIMO recently was published. The most intriguing features of PRIMO are the user-friendly approach, the stand-alone property and the built-in definition of different linear accelerators models. Nevertheless, IMRT simulations are not yet implemented.

**Methods:**

A Varian Trilogy with a Millennium120 MLC and a Varian Novalis with 120HD MLC were studied. A RW3 multi-slab phantom was irradiated with Gafchromic films inserted between slabs. An Expression 10000XL scanner (Seiko Epson Corp., Nagano, Japan) was used to digitalize the films. PTW-Verisoft software using the global Gamma Function (2%, 2 mm) was used to compare simulated and experimental results.

The primary beam parameters were adjusted to best match reference data previously obtained in a water phantom. Static MLC simulations were performed to validate the MLC models in use. Two Dynamic IMRT preliminary tests were performed with leaves moving with constant and variable speed. A further test of an in phantom delivery of a real IMRT field allowed simulating a clinical-like MLC modulation.

**Results:**

Simulated PDD, X- and Y-profiles in reference conditions showed respectively 100.0%, 100.0% and 99.4% of Gamma points < 1 (2%, 2 mm). Static MLC simulations showed 100.0% of Gamma points < 1 with the 120HD MLC and 99.1% with the Millennium compared with the scanned images.

The fixed speed test showed 99.5 and 98.9% of Gamma points < 1 respectively with two different MLC configuration-sampling algorithms when the 120HD MLC was used. The higher modulation MLC motion simulation showed 99.1% of Gamma points < 1 with respect to the experimental. This result depends on the number of the fields to reproduce the MLC motion, as well as calculation time. The clinical-like simulation showed 96.2% of Gamma points < 1 using the same analysis conditions.

**Conclusions:**

The numerical model of the Varian Trilogy and Novalis in the PRIMO software was validated. The algorithms to simulate MLC motion were considered reliable. A clinical-like procedure was successfully simulated.

## Background

Intensity-Modulated Radiation Therapy (IMRT) [1–4] is an External Radiotherapy advanced technique, nowadays considered as one of the standard Radiotherapy (RT) treatment modalities. It is generally able to provide a higher dose conformation to the target and significantly higher dose sparing to surrounding tissues than conventional treatment methods such as 3D conformal RT (3DCRT). This superior treatment modality needs a dedicated quality assurance (QA) program to ensure the safety of patients and to minimize the uncertainties associated with the procedure. Examples of potential error sources are target location, patient setup uncertainties and organ motion during the irradiation. Furthermore, the Treatment Planning System (TPS) dose calculation algorithms introduce inaccuracies due to the necessity of simplification of the models for radiation interaction in the tissues, in order to reduce the calculation time. In general, a comprehensive QA in advanced RT should require patient-specific dose verifications.

Different QA measurement techniques in IMRT exist, making use of 2D detector array [5–8], single ion chamber in phantom for point dose measurements [9–11] or using specific phantoms with 2D dose measurement devices and 3D dose reconstruction software [12–14]. One disadvantage of these approaches is that measurements are generally compared with calculations by the TPS and it is difficult to give interpretation and to address the deviations between calculated and measured doses to failures of the accelerator performance or to the calculation algorithm. Also, the dose calculation is generally performed in a homogenous phantom and often a single QA measurement does not necessarily provide direct information on the dose distribution in the patient during treatment delivery. Furthermore, spatial resolution is a limitation given by the finite distance between the detectors in dose verification devices.

In Medical Physics, several dosimetric problems have been approached by the Monte Carlo (MC) method [15]. MC approach is considered to be the gold standard method [16–19] and in some cases the only one, to perform reliable absorbed dose calculations because it provides the most detailed and complete description of the radiation fields and of the particle transport in tissues. MC method can be used to numerically simulate the irradiation by introducing geometrical and physical information in dedicated computer software. From this point of view, Monte Carlo (MC) is a powerful method to be included in a comprehensive QA program of IMRT and VMAT as it allows accurate determination of 3D dose distribution description in both phantom and patient setup and the numerical solution can provide comprehensive information for RT treatment QA. MC simulation can assist to understand eventual discrepancies between measured and calculated dose and allow determining if a machine failure or dose miscalculation occurred. Also, MC simulations can give information about 3D dose and visualize the results in both homogeneous and inhomogeneous phantom as well as in a patient 3D model. In addition, a solid and robust MC code can accurately calculate dose in critical conditions where the TPS is known to suffer poor calculation accuracy and point out any TPS dose miscalculation.

Several codes are available for simulation in the field of RT, such as GEANT4 [20], EGSnrc/BEAMnrc [21], PENELOPE [22], FLUKA [23] and MCNP [24]. Recently, a new MC code named PRIMO that makes use of the PENELOPE features was developed [25]. The PRIMO simulation software has a user-friendly approach, which is a suitable and competitive characteristic for clinical activity. Different linear accelerators (LINAC) models and Multi Leaf Collimators (MLC) components are provided in the PRIMO release, such as Varian Clinac 2100 and Varian Clinac 2300 and the Millennium 120 and 120HD MLC. Nevertheless, advanced features such as IMRT simulations are not introduced yet in PRIMO.

MC simulations of MLC-based of both step-and-shoot and dynamic IMRT procedure have been tackled by different authors. Ma et al. [26] used the particle dependent weighting factor method, applying different weights to each particle according to the integral linear attenuation encountered by a ray passing through the beam modifiers. Leal et al. [27] and Seco et al. [28] adopted the full MC simulation strategy, simulating the particle tracking through all the components of the unit and implementing the Static Component Simulation (SCS) as described by Shih et al. [29] to reproduce a step-and-shoot IMRT delivery. Liu et al. [30] firstly described the Position-Probability Sampling (PPS) method, which faces the unit component motion, such as the MLC leaves, from a probabilistic point of view. Heath and Seuntjens [31] adopted a similar strategy into BEAMnrc.

PRIMO is stand-alone software, which does not need any code written by the user to be fully configured and run. On the other hand, it does not include advanced tracking features. Nevertheless, PRIMO allows multi-beam simulations, with different geometric setup for each single beam. This feature can be used to implement both the SCS and PPS strategy to reproduce the MLC beam modulation in both step-and-shoot and dynamic IMRT mode.

The clinical implementation of IMRT MC simulations requests robust, reliable and fast results. MC simulations are well known to be time consuming, which can be unsuitable for the clinical activity. The calculation time of a MC simulation depends on a very large number of parameters, such as the number of primary histories, the requested uncertainty, the use of variance reduction algorithms, and the characteristics of the hardware. The IMRT MC simulation, performed dividing the dynamic procedure in static fields, introduces the number of fields as further degree of freedom in the simulation setup. As stated by Seco [17], the number of particles to be tracked, and the time dedicated to particle transport simulation, does not depend on the number of fields in which the procedure is split, while a higher number of static fields better approaches the behaviour of a continuous motion.

The aim of the present paper is to describe the configuration and use of PRIMO to simulate an IMRT procedure and the results of a preliminary feasibility study on whether it is possible or not to use it to perform an IMRT simulation.

## Methods

In this paper we present the results of a preliminary feasibility study of PRIMO MC simulations of IMRT procedures on Varian RT units. This study particularly focuses on the simulation of two specific RT units, both equipped with Varian 2300IX LINAC head: a Varian Trilogy using Millennium120 as MLC and a Varian Novalis mounting 120HD MLC.

The 2300 LINAC head is incorporated in PRIMO software as one of the available models, as well as both the Millennium120 and 120 HD MLC systems. The considered RT units are able to produce 6, 10, or 15 MeV (6, 10 or 15MV photon beams) beams, but for this study, only the 6 MeV (6MV photon beams) beam has been used.

Specific IMRT procedures, for in phantom dose measurements, were planned making use of the Varian Eclipse TPS. The simulations were performed on a Intel(R) Xeon(R) CPU E5–2660 v3 @ 2.60GHz 2.60GHz with 16GB of RAM, with 32 CPU cores available, but only with a maximum of 30 working simultaneously. The version of PRIMO installed is 0.1.5.1307 downloaded from https://www.primoproject.net.

The phantom used for the measurements was a multi-slab RW3 box with SSD 95 cm. EBT3 Gafchromic films from a single batch and cut as a 15 × 15 cm^2^ square were inserted between two slabs at 5 cm depth in the phantom to allow comparison between simulations and experimental dose distributions. Dose distribution images were obtained by scanning the Gafchromic film with an Expression 10000XL scanner (Seiko Epson Corp., Nagano, Japan) and using a calibration curve of 17 points, from 10 to 500 cGy, obtained in reference conditions (10 × 10 cm^2^ field, SSD 100 cm and the films positioned 5 cm deep from the phantom surface) for a 6 MV photon beam. The calibration films were digitized 48 h after irradiation.

### PRIMO software

The PRIMO software is a graphical user interface based on the PENELOPE 2011 computational engine, which, providing the geometrical and physical models of most Varian and Elekta LINACS, facilitates the MC simulation of these RT units. The PRIMO software divides the simulation process into three steps, hereby referred as the *s1*, *s2* and *s3* (following the PRIMO nomenclature). The s1 + s2 stages represent the LINAC head simulation. The *s1* is the patient independent stage, from the primary electron beam, hitting the target, to above the jaws. The correct simulation stages include the tuning of the primary beam parameters, in order to obtain agreement with a set of measurement data under specific conditions. The patient dependent *s2* stage is the simulation of the particles passing through the collimation, from above both the jaws to below the MLC systems. Both the *s1* and *s2* steps provide an IAEA formatted phase space file as the output. The *s1* phase space file (*phsp1*) contains information about particles leaving the LINAC head, while the *s2* phase space file (*phsp2*) describes the beam particles after interaction with the collimation system. The s2 stage uses the phsp1 as the radiation source, while the last stage, *s3*, tracks the *phsp2* particles in exit from the collimation system, into the phantom. As the output of this stage, the 3D dose distribution is obtained in a specific PRIMO formatted shape.

### LINAC heads simulation

The simulation of the LINAC head (*s1*) was firstly performed comparing the results with dosimetric data obtained in a water phantom (MP3 phantom tank) with a Semi-flex Thimble Chamber with 0,125cm^3^ volume (PTW-Freiburg, Germany). PRIMO defines the energy distribution of the primary electrons hitting the target as a Gaussian distribution with the center of the distribution E_mean_ and the Full Width at Half Maximum E_FWHM_. These parameters affect the Percentage Depth Dose (PDD) of the simulated radiation beam. The software suggests default values for E_mean_ and E_FWHM_ of the distribution, which were tuned in order to obtain agreement with experimental PDD in a trial and error approach. The software offers further parameters, to take into account the dimension of the area where the primary electrons hit the target (focal spot) and the beam divergence. These have slight influence on the PDD, but strongly determine the spread of particles, and, consequently, were adjusted to match the experimental lateral dose profiles. The *s1* stage simulation was validated, through comparison with experimental PDD and Off-axis dose profiles measured in reference conditions in a water phantom: beam size 10 × 10 cm^2^ at isocenter and Source Surface Distance (SSD) 100 cm. The beam parameters were adjusted until the agreement with the experimental dataset was acceptable according to the Gamma Function analysis [32], adopting global, 2%, 2 mm as the Gamma parameters, and 95% of Gamma points < 1 as the passing rate in every case. Preliminary tests were performed to verify the balance between number of histories, voxel size in the phantoms, uncertainty of the dose value in the voxels and usage of variance reduction algorithms. The requirement for this step was to achieve uncertainty of 1% for voxels with dose values greater than 50% of the maximum value and the s1 stage was stopped once this condition was reached. After, the phsp1 was fully used as the radiation source for the following stages, simulating all the particles collected in it, by setting to reach the total histories number as the stopping condition.

### Static MLC simulations

Once the primary beam parameters were adjusted and the *phsp1* was obtained, both the *s2* and *s3* stages were simulated with the insertion of a static configuration of the MLC for validation purpose. The PRIMO was configured using *phsp1* as the particle source. Two different simulations in static MLC configuration, one for each unit, were set up. The static configuration was defined to obtain a sequence of open/closed leaves in a definite pattern. In particular, three groups of leaves were left open: a) a first group with three adjacent leaves, b) another group with just two leaves and c) a single leaf open. The three groups were located in the central area of the radiation beam. Both simulated and experimental data were acquired.

The PRIMO was configured by introducing the leaves position values into the input file. This approach can be time consuming and prone to errors, especially if more than one single field is to be simulated, because one value per leaf (120 in this case) per field must be typed. The simulation geometry and materials were defined as the same of the routinely QA measurements: solid water (RW3) phantom and 95 cm as the SSD.

The experimental irradiation was executed in phantom as previously described with the requested MLC configuration beams.

### Dynamic MLC simulation

The MLC geometrical and physical model validation was compulsory to approach the simulation of IMRT procedures, since the radiation intensity modulation is performed using the MLC as a beam modifier. Two different IMRT modalities can be used: step-and-shoot or dynamic. While the step-and-shoot IMRT can be essentially simulated as a series of static fields, the dynamic modality poses the problem of how to reproduce a continuous event in a computerized system, which, by its nature, works by discrete states. Since the aim of this work is to simulate a generic IMRT treatment, an approach to simulate the dynamic IMRT making use of PRIMO was studied. The SCS [24] method is suitable to simulate a step-and-shoot IMRT, while the PPS [25] is a dynamic simulation strategy as described before.

According to both the SCS and the PPS approach, the simulation of the movements of the MLC is performed by dividing the whole process in a number of discrete configurations of the MLC. The normalized cumulative fraction of the total Monitor Units (MU) of a dynamic procedure is named as the MU_index_. MU_index_ spans between *0.0* and *1.0*. The trajectory of each single leaf in MLC can be represented as a function of MU_index_. A control point is defined as the MLC configuration at a determinate MU_index_. In order to implement the IMRT simulation, the basic information on how the MLC moves during the beam-on is required.

Two different objects can provide information on the MLC movements and were used in this work to reproduce the MLC configuration motion during the treatment.

1. Varian Multi Leaf Collimator *.mlc* file, produced by the Eclipse TPS (MLC file)
2. DynaLog file of the MLC, after the irradiation

A number of tools were developed during this project to open, read and redesign the information type of both these files. A number of control points define the MLC bank trajectory, assuming that the leaves move continuously between consecutive control points. A specific tool was developed to allow interpolating the MLC configuration at specific MU_index_ values. Both the SCS and the PPS strategies are implemented using the .mlc and the DynaLog files as the sources of information. In the first case, as the .mlc file is generated by the TPS, it means to prospectively simulate the plan and to obtain the planned dose distribution. Assuming the MC code to be absolutely reliable, the comparison with the experimental data can highlight possible incorrect performances of the LINAC during the treatment. On the other hand, the DynaLog is a source of retrospective information and a MC IMRT simulation based on it intends to reproduce the actual MLC motion. This approach can help to individuate the cause of machine failure and its dosimetric consequences on the patient.

#### Multi leaf collimator file (MLC file)

In IMRT, the TPS calculates the MLC modulation to achieve the expected fluence of particles in order to satisfy the clinical requirements. The Varian systems report this information in a specific *.mlc* formatted file, with a header and a body. The header contains general information on the treatment such as the patient name, the RT unit, the MLC in use. The planned configuration of the MLC during the delivery is described in a specific format shape, by a number of control points, the first being at MU_index_ = 0 and the last at MU_index_ = 1.

#### DynaLog file

The *DynaLog* files can be considered as a retrospective source of information on the MLC configuration as a function of MU_index_. Every 50 ms, the system performs an internal check on the positions of each leaf and saves it in a file available at the end of the irradiation. The data is stored in an MxN matrix shape, where N is the number of leaves of the MLC and M is the number of the positions checks performed by the system. By reading the *DynaLog* matrix, the MLC trajectory is reconstructed.

### PRIMO output manipulation

Some output data manipulation was required to validate the simulations results of the MLC model with respect to the dose digitalized image acquired by the Gafchromic film. The aim of the data manipulation was to perform a direct comparison between simulated and experimental 2D dose distributions. A powerful tool that allows a 2D Gamma Analysis commonly used in Medical Physics Departments is the PTW Verisoft analysis software. This software accepts dose images as input, in a *Tiff* or *DICOM* format file. The scanned Gafchromic dose images are in the *Tiff* format, while the PRIMO output file is a sequence of dose values, one per voxel. Consequently, an *in-house* code was developed to manipulate the PRIMO output in order to reshape the data in a volumetric 3D matrix form and to select specifically located data (e.g a 2D dose plane), to create dose images and save those in *DICOM* format. Following this procedure, as a final result of this stage, 2D Gamma Analysis compared the experimental dose image acquired with the Gafchromic film and the 2D dose image simulated at the film location. This data rearrangement was also applied on all the simulations hereafter described, static and dynamic, for both the Millennium120 and the 120HD MLC models.

### PRIMO MLC dynamic configuration file

The PRIMO software requires a specific configuration file (*.ppj*), which contains a number of static fields information, including the position of each leaf for each field. The definition of the MLC arrangement can be performed visually in PRIMO by selecting, dragging and dropping the leaves or, alternatively, by typing in the *.ppj* file. The definition of the *.ppj* file is not straightforward when a large number of fields are to be simulated and to write the file *by hand* is not a feasible solution. Furthermore, the simulation of a large number of fields can present a great computational effort if a hard post-simulation processing stage is required, incompatible with the clinical implementation of the IMRT MC simulations. For this reason, a relatively low number of static fields is preferred as a compromise, interpolating the MLC configurations only at specific *Control Points*.

An *in-house* code was written to automatically read the information from the MLC configuration source (*.mlc* or *DynaLog* file) and provide the correspondent *.ppj* file to be correctly interpreted by PRIMO. Two different algorithms were developed to configure PRIMO, according to two different methods of sampling the MLC configuration as a function of MU_index_.

#### Fixed step sampling

A first straightforward choice of the *Control Points* to configure PRIMO to simulate IMRT procedure is by dividing the whole procedure in a set of N intervals, separated by fixed gap in MU_index_. The input file (*.mlc* or *DynaLog*) can be interpolated at a fixed step of MU_index_. Every static field configured represents a fraction of 1/N of the total MU.

This approach ensures that the *Control Point* values are sampled uniformly all along the whole procedure and implements the SCS idea. Unfortunately, this proposed algorithm, although simple, can introduce a sampling pattern in the selection of the MU_index_ values. One solution to overcome this problem is to randomly sample the MLC motion by creating a random array of *Control Points.*

#### Random sampling

A more sophisticated method to define the *Control Points* in order to describe the motion of the MLC, implements the SCS by randomly sampling intermediate positions. This algorithm avoids any possible pattern in the choice of the *Control Points*, and allows a higher degree of fidelity in the simulation of the continuous dynamic motion of the MLC as stated by Liu et al. [30]. Nevertheless, this approach introduces a non-uniform sampling of the procedure. Each single static field configured represents a different fraction of MUs, as the MU_index_ separation between adjacent *Control Points* is not fixed. Accordingly, a different weight must be given to each field.

### MC simulation of a MLC modulated delivery

After implementing the different codes to configure PRIMO, to simulate dynamic MLC procedures, to analyse data and to create 2D images of simulated dose distribution at specific planes, a couple of basic simulations of IMRT were performed.

The first test aims to give an answer to whether it is possible or not to use PRIMO to simulate the movements of a leaf sliding with constant speed during the whole delivery. Actually, three groups of adjacent leaves were left free to slide. The first group was composed of three leaves referred as the numbers 25, 26, 27 of the A bank side, while in the second group the 30 and 31 leaves of the A bank side and in the third group the single 36 leaf of the A bank were configured to move with fixed speed.

The simulation was performed using 100 interpolated positions with both algorithms to configure PRIMO: the fixed step as described in subsection 2.6.1 and the random sampling as described in subsection 2.6.2.

The same setup used for the simulations, was adopted experimentally in order to allow comparisons. The film was digitalized and the image used as the reference for simulation result evaluation.

**Fig. 1:**
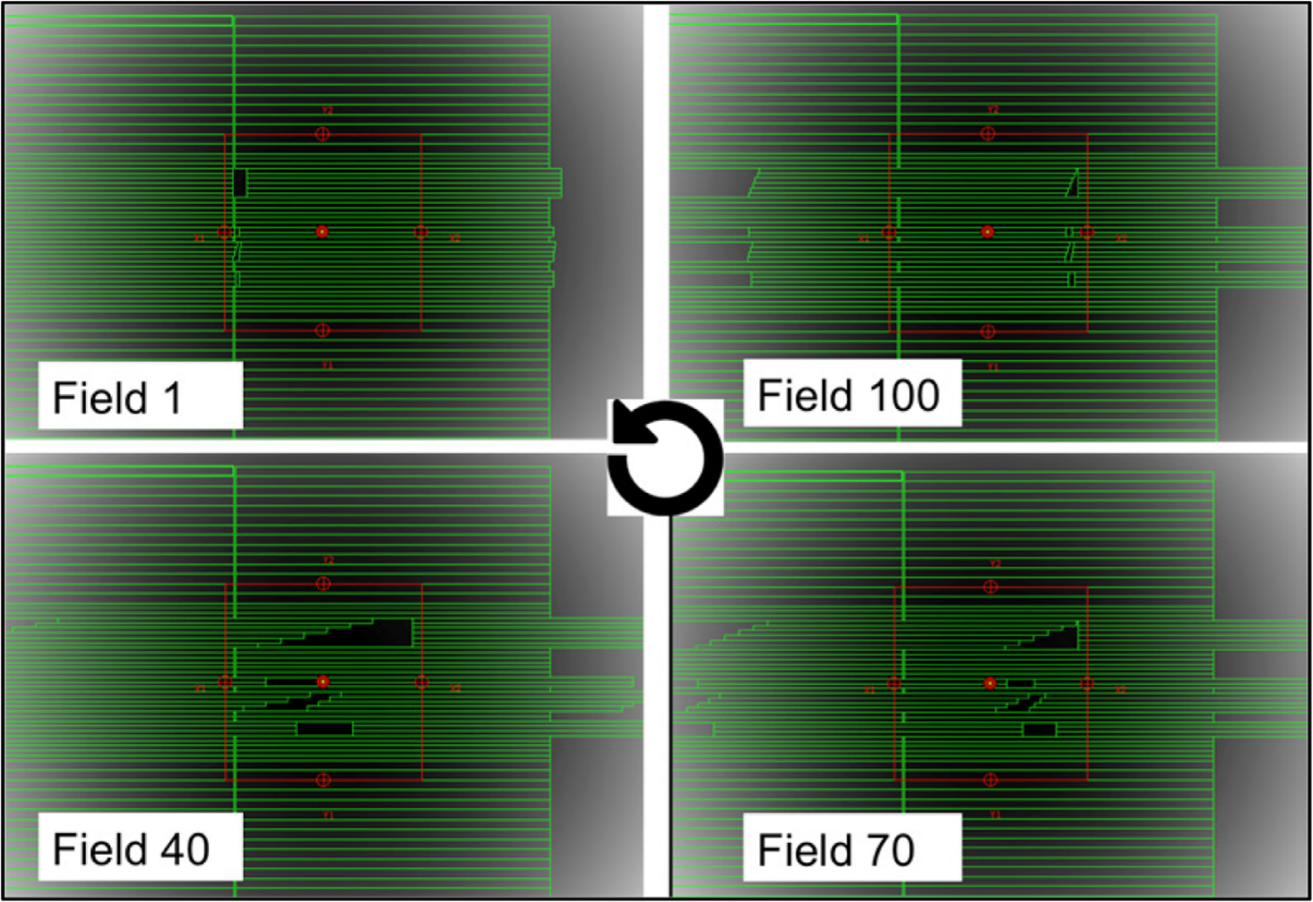
Example of higher modulation dynamic procedure divided in 100 static fields

A second test was performed in analogous configuration as the first test, with higher MLC modulation, including leaves acceleration during the procedure and different speed between adjacent leaves, resulting in a dose pattern with higher gradient. The MLC motion is shown in Fig. 1. The simulation was performed with 100 fields randomly interpolated by the MLC motion in the same setup of the irradiation.

To evaluate the right balance between number of fields and calculation time, this IMRT procedure was repeated using different numbers of fields, from 2 to 180 and the gamma function was used to assess the quality of the simulation as a function of the number of fields. The separate *s2* and *s3* calculation time was registered for each condition and reported as a function of the number of fields in use.

### MC simulation of a clinical-like MLC modulation

A third test was performed in a more complex situation. A real IMRT field of a prostate treatment, as calculated by the TPS on a real patient was considered. The field showed a complex modulation of MLC. The field was delivered using the same setup of the previous case. Once the film was irradiated, the *DynaLog* file of the procedure was exported. The *DynaLog* file was used to configure PRIMO and to perform a simulation by random sampling 150 MLC configurations during the whole delivery. A second simulation was configured including the whole set of 272 MLC positions showed in the *DynaLog* file. Since PRIMO allows simulating only 180 filed at once, the whole simulation was split in two parts, each one with 136 static fields. As in the other cases, the 2D Gamma analysis was performed to validate the results.

## Results

### Stage *s1* simulation

Using the *phsp1* as the primary beam source in reference conditions, allowed obtaining sufficiently low statistical uncertainty (around 1%) in a voxelized water phantom, with voxel size of 0.1 × 0.1 × 0.2cm^3^. The total number of primary electrons used in the *s1* stage was around 2.1*10^8^, for a total calculation time of approximately two weeks, activating the splitting factor of 200.

As described in section 2.2, the first step of this work was to tune the beam parameters according to a set of experimental data. The best choice of the parameters is:

- Primary electrons mean energy: 5.9 MeV
- Primary electrons FWHM energy: 0.2 MeV
- Primary electrons focal spot: 1.5 mm
- Primary electrons beam divergence: 0.1°

**Fig. 2:**
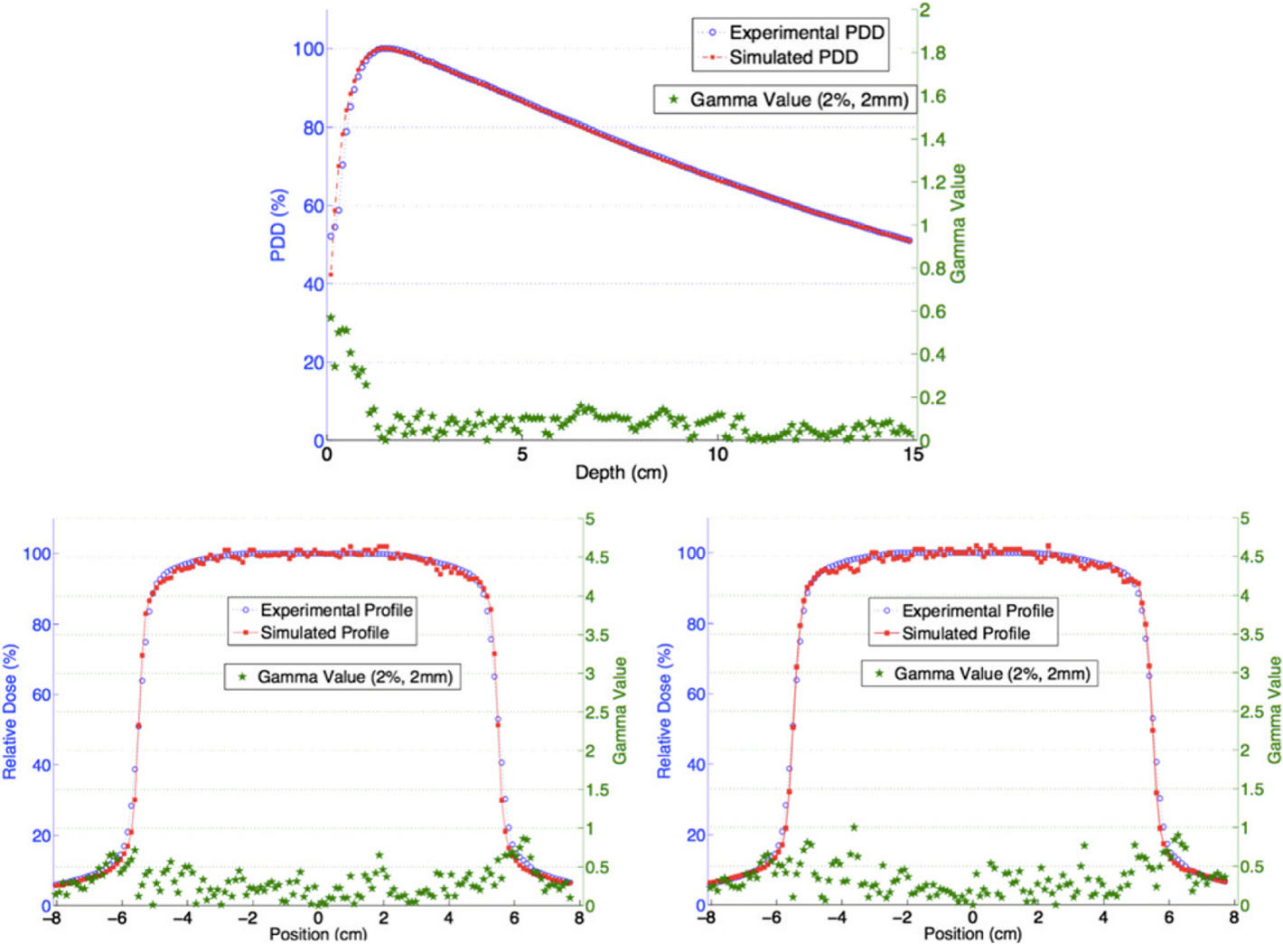
Experimental (red) and simulated (blue) PDD (top), X-profiles (left bottom) and Y-profiles (right bottom). The green data represent the Gamma values reported according to the right vertical axes

These parameters were consistent for the LINAC head of both units considered in this work. The results are graphically reported for the PDD and both X- and Y-profiles in Fig. 2, where blue data represent the experimental set acquired on the Trilogy unit and red points are the simulated. The green stars refer to the right vertical axes and represent the result of the Gamma analysis.

The number of valid Gamma points (2%, 2 mm) confirms the good agreement between simulations and experimental measurements. The PDD showed 100.0% of Gamma points < 1. While the dose profile in the X direction, at 10 cm depth in the water phantom shows 100.0% of Gamma points < 1, the profile in the Y direction showed 99.4%.

It is important to note that the same *phsp1* was used as a particle source for both the RT unit considered. The same simulated data showed comparable good agreement with respect to the data of the Novalis unit. The LINAC model is considered validated for both the unit used in this work.

### Static MLC simulation

**Fig. 3:**
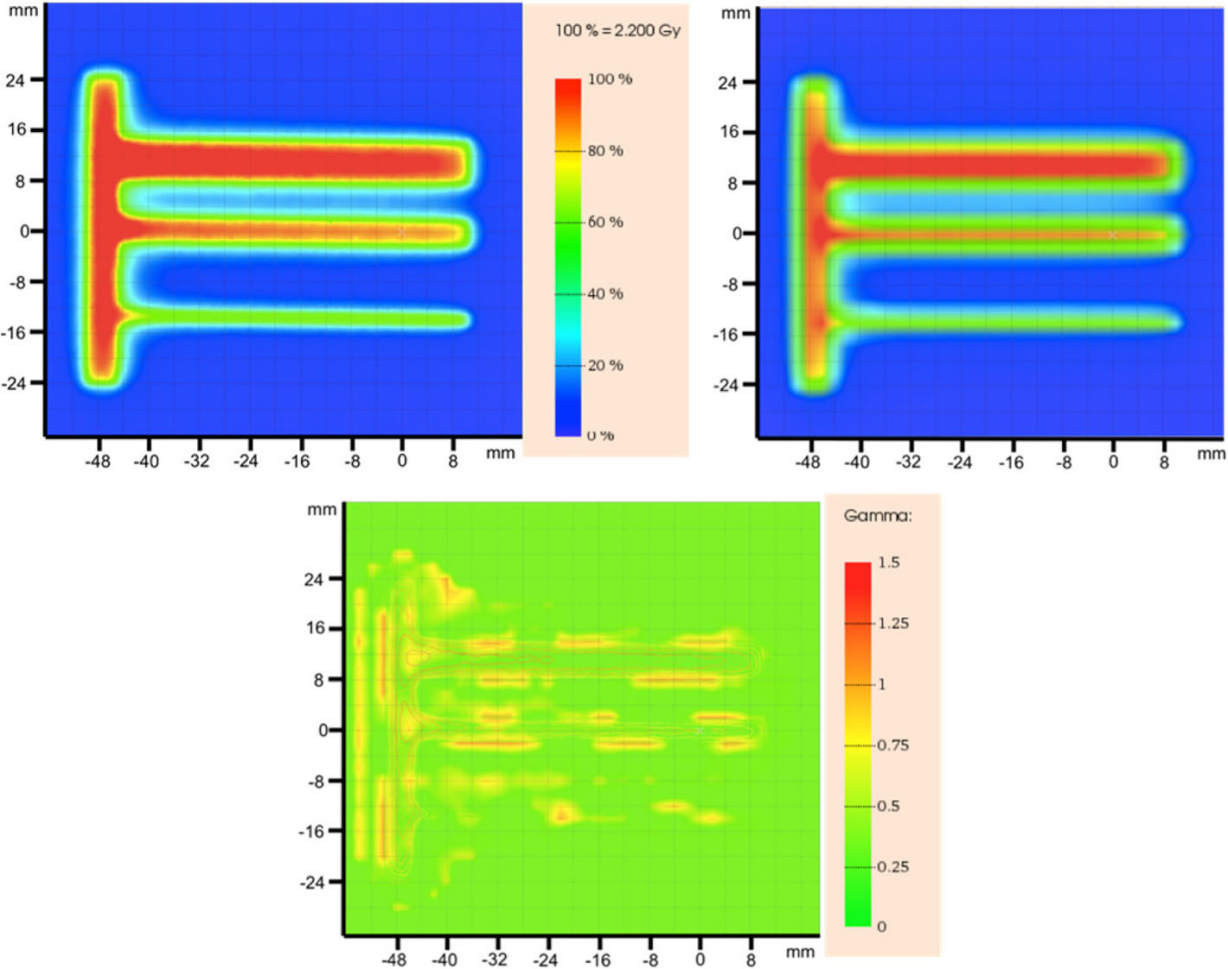
Dose distribution comparison between experimental data as acquired by the Gafchromic film (top left) and the simulated data (top right) for the static delivery with 120HD MLC in use. The (2%, 2 mm) evaluation showed 99.1% of gamma points lower than 1. On bottom, the gamma values distribution. PTW Verisoft was used to calculate the gamma values

The *phsp1* file was used as the beam particle source for the static simulation of a 10 × 10 cm^2^ field with static 120HD MLC configuration inserted. The comparison between the experimental dose 2D image and the simulated at 5 cm depth in the solid water phantom confirms that the 120HD MLC model used in this work is reliable. In Fig. 3 the Gamma value distribution is reported. As 100.0% of Gamma points < 1 were observed, the 120HD MLC model is considered validated. The same analysis was performed when the Millennium 120 MLC was in use, obtaining 99.1% of Gamma points < 1. Also, this MLC model is considered validated.

### Dynamic MLC simulations

**Fig. 4:**
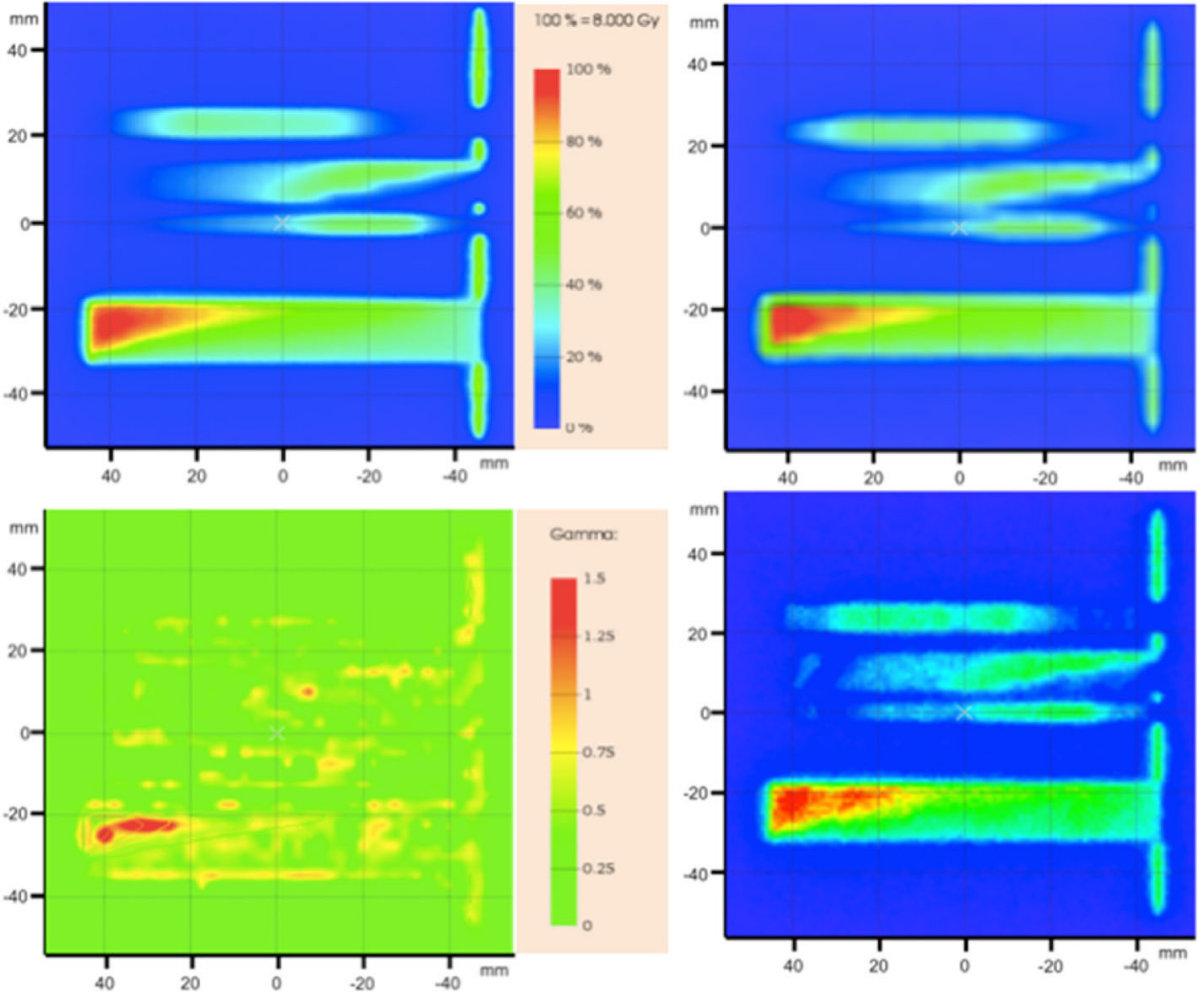
Dose distribution comparison between experimental data as acquired by the Gafchromic film (top left) and the simulated result using 100 random static fields (top right) for the high modulation dynamic delivery described in section 3.3. The 2%, 2 mm evaluation (left bottom) showed 99.5% of gamma points lower than 1. PTW Verisoft was used to calculate the gamma values. On bottom right the dose distribution at the film location when 20 fields are used

As described in section 2.7, a first basic test to simulate leaves moving with constant speed was performed by sampling 100 static MLC configurations with the fixed step method. The agreement is confirmed by the 99.5% of Gamma points < 1, comparing the 2D dose distribution at the film location with respect to the Gafchromic digitalized image considered as the reference. The same simulation was repeated using the same number of static fields, but randomly sampled. The Gamma analysis in this case shows 98.9% of Gamma points < 1. A direct comparison of the dose image at the film location between the two sampling methods results in 99.8% of Gamma points < 1. Similar results were obtained in an analogous case when the Trilogy unit with the Millennium 120 MLC was used. A more complex MLC motion, described in section 2.7, was simulated and compared with the Gafchromic film. Figure 4 shows the Gafchromic digitalized image (top left) and the 2D image of the dose at the film location simulated with 100 fields (top right) and 20 fields (bottom right) randomly generated. A first visual inspection shows a good agreement when 100 fields are used, which is confirmed by the 99.1% of Gamma points < 1 obtained from the gamma analysis. The distribution of the Gamma points is shown in the Figure (bottom left). On the contrary, the discretization with a lower number of fields appears as a worse simulated result compared with the experimental data. The simulated dose distribution at the film location when 20 fields are simulated is shown in Fig. 4 (bottom right), resulting in 75.0% of the gamma points < 1.

### Simulation of a clinical MLC configuration

**Fig. 5:**
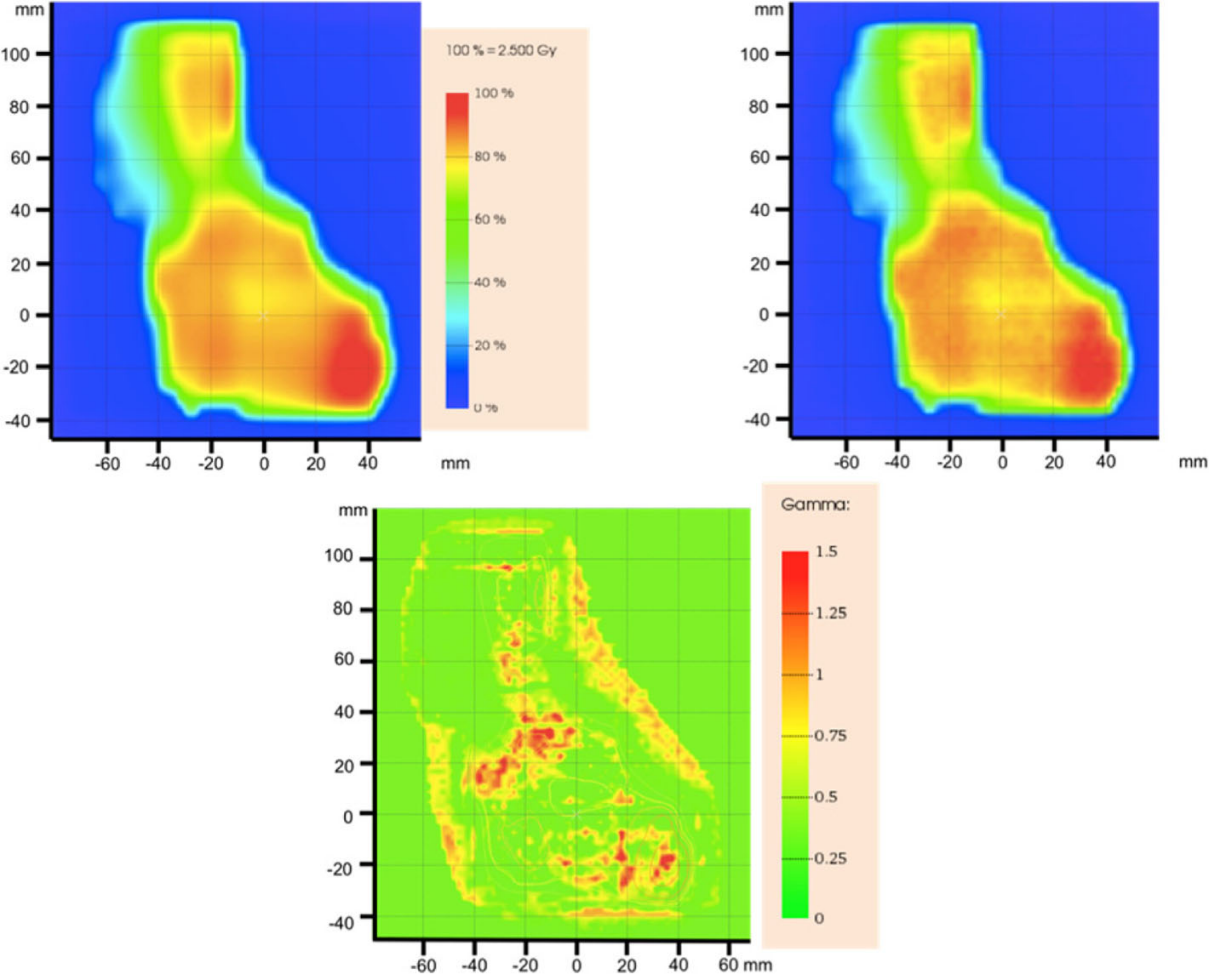
Dose distribution comparison between experimental data as acquired by the Gafchromic film (top left) and the simulated result using 150 random static fields (top right) for the IMRT dynamic procedure of real patient delivered in phantom. The 2%, 2 mm evaluation (left bottom) showed 96.2% of gamma points lower than 1. PTW Verisoft was used to calculate the gamma values. On bottom the 2D distribution of the Gamma values

Figure 5 shows the comparison between experimental and simulated dose when 150 randomly sampled MLC configurations are used to represent the dynamic motion of the MLC. On top left, the experimental dose distribution as measured by the Gafchromic film and, on top right, the simulated dose distribution at the film location. Quantitatively, the Gamma analysis in this case shows 96.2% of points < 1. The distribution of the Gamma points is also shown in Fig. 5 (bottom). The result of the further simulation, performed with all the 272 MLC configurations present in the *DynaLog* file, shows 95.5% of the Gamma points < 1. A direct comparison between the two cases reveals 99.6% of Gamma points < 1.

### Approaching methodology for simulation time optimization

**Fig. 6:**
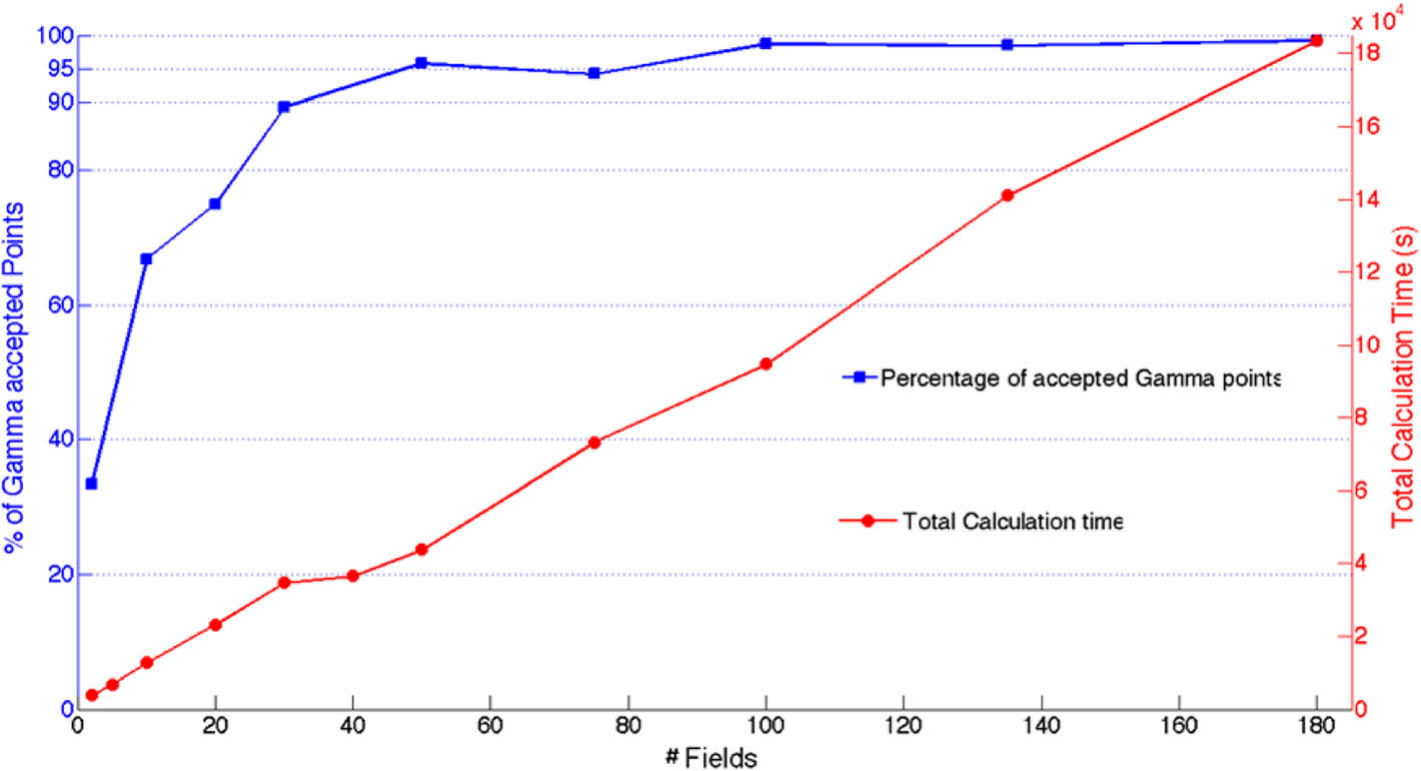
Percentage of Gamma points accepted (blue) and s2 + s3 calculation time (red) for the simulation described in section 2.7, repeated dividing it in a different number of static fields

The Gamma approach was used to evaluate the simulation of the IMRT procedure described in the section 2.7 when using different number of static fields. The percentage of accepted points is reported in Fig. 6 as a function of the number of the static fields used to reproduce the MLC motion in comparison with the total calculation time of the collimation (*s2*) and dose deposition (*s3*) stages. When the number of static fields used reduces down to a minimum of 2, the fraction of Gamma points < 1 is 33.5%, indicating that not enough discrete positions are sampled. By increasing the number of newly randomly sampled static fields, the dose distribution approaches the measured one, as indicated by the increasing percentage of Gamma points < 1. When the simulation is divided into 20 static fields, 75.0% of the Gamma points are < 1. The minimum fraction of accepted Gamma points to validate the simulation (95%) is reached when at least 50 fields are used. Beyond this value, an increase in the field number does not improve significantly the quality of the simulation with respect to the experimental data, and the accepted points asymptotically tend to 100%.

On the other hand, the total calculation time to simulate the collimation and the dose deposition in the phantom increases linearly with the number of fields. In Fig. 6 it is shown that, using the maximum number of CPUs (30), the *s2 + s3* calculation time increases from the minimum value of 63 min to simulate two fields, to 51 h in the case of 180 fields in use. It is important to underline that these results are relative to this specific IMRT simulation and are not intended to be general, although a similar behaviour should be expected.

## Discussion

### Validation of PRIMO IMRT simulations

PRIMO provides a model for the Varian 2300IX LINAC head as well as for the 120HD and Millennium120 MLC. The validation of the beam parameters with respect to the experimental data was compulsory to create a phase space as a radiation source and to simulate dynamic MLC procedures. The LINAC head models resulted valid as more than 95% of Gamma points were < 1 in PDD and dose profiles in water tank. In addition, both the MLC models were assessed as 100.0 and 99.1% of 2D Gamma points for the static simulations described in 2.3 were accepted respectively with 120HD and Millennium120 in use. This result has a twofold importance. On one hand, since the radiation beam is modulated by the MLC, the good quality of the MLC models is requested to guarantee reliable MC simulations. On the other hand, this result represents a novelty, because the two MLC models in PRIMO had never been checked before, with respect to experimental data. This result improves the confidence in the PRIMO software as a reliable tool for MC simulation tool in Radiotherapy.

Two algorithms were used to automatically configure PRIMO to simulate an IMRT procedure: the fixed step creates static MLC arrangements with a constant gap in terms of MUs between the fields, while a second algorithm randomly samples the MLC configurations. A first basic test was performed to evaluate both the algorithms to reproduce a fixed speed motion of the MLC. The fraction of accepted Gamma points was higher than 95% in both cases validating the two algorithms and the small difference between them proves that, in the specific case of 100 static fields, the algorithms are equivalent.

The randomly sampling algorithm was used further, performing a second test to evaluate the algorithm in the case of complex MLC modulation, especially in the case of leaves acceleration and deceleration and different speed between adjacent leaves. This case, simulated on the Novalis unit dividing the procedure in 100 fields, reported 99.1% of Gamma points < 1 with respect to the experimental Gafchromic reference, proving the reliability of the algorithm with sufficiently high number of static fields, even in a highly modulated condition.

These initial studies posed the basis for a deeper investigation on the capability of PRIMO to simulate IMRT treatments, with the final aim of the clinical implementation.

After validating the static LINAC head, the MLC components and the dynamic simulation algorithms, a test similar to a real clinical case was performed. A prostate IMRT field was delivered on a multi-slab RW3 solid water phantom in the common pre-treatment QA setup with Gafchromic film. The DynaLog of the delivery, composed by 272 measurements, was used to create the static fields to be simulated by PRIMO. The tool developed in this work, using the DynaLog file as the input to automatically configure MC simulations, can be very useful, because it allows quantitative dosimetric verification of real IMRT deliveries in patients and a retrospective verification in the QA program, by simulating the actual IMRT procedure. The simulation and the irradiation described in 2.8 were performed in phantom, and a direct comparison showed 96.2% of Gamma points < 1 in the evaluation of 2D Gamma when only 150 fields were randomly interpolated. Increasing the number of fields up to 272, does not improve the agreement of the simulated results with respect to the experimental data.

### Simulation quality and calculation time relation

The simulation with higher modulation described in section 2.7 was repeated using a different MU average resolution. That is: dividing it in different numbers of static fields. When the number of static fields is reduced, the simulation does not match the experimental data as shown by the case of 2 to 50 static fields used. As an example, when just 2 fields are simulated, the dose distribution at the film location is not matching the experimental as only 33.5% of Gamma points are < 1. This is expected, since 2 frames cannot well describe a complex dynamic motion. Conversely, when 50 fields are simulated, 95.9% of Gamma points < 1, passing the acceptance criteria. This highlights that a minimum number of static fields is requested to satisfactorily simulate an IMRT procedure. The best simulation arrangement is obtained when the maximum number of fields (180) is configured, as 99.5% of the Gamma points were < 1.

As a counterpart, the improvement of the MU resolution is accompanied by an increment of the calculation time. In Fig. 6, the calculation time can be assumed linearly dependent on the number of fields. This result opens to a twofold discussion. On one hand, increasing the number of fields beyond a specific number, results in a very small improvement in the simulation quality. 50 fields give 95.9%, while 180 fields rise to 99.3% of Gamma points accepted. Consequently, increasing indiscriminately the number of fields results in a time/quality inefficient process. On the other hand, the time increment is quite unexpected. As stated by other authors [14], the calculation time for different fields should in principle be independent on the number of the geometries to be simulated. This rationale, in the specific case of IMRT MC, is driven by the assumption that the time to simulate the collimation and dose deposition only depends on the number of particles in the source *phsp*. The requirement for this rational to be true is that the pre-tracking configuration and the post-processing time is negligible in comparison with the tracking time. If this condition is not satisfied, unexpected results will arise. In particular, the PRIMO workflow per each field begins with reading the materials cross sectional data matrixes and the geometry definition in the simulation. These processes can take up to some minutes, and during these steps, the particle tracking is not running. This time can become relevant when several fields have to be simulated as in the cases described in this work. In addition, PRIMO works with text files to store the dose distribution information. At the end of every single static field, a post-processing operation involves summing up all the dose distributions from every parallel process running simultaneously. This operation requires accessing, reading, calculating and writing data for each static field and it results in a time consuming operation. Especially, this result is true when these steps must be repeated several times, as it is the case of a multiple field simulation. Also, it can be argued that the number of data to access, read and write could influence the post-processing time. In other words, the number of voxels is expected to be a crucial parameter in the calculation time optimization.

### Observations toward the clinical implementation

These tests were performed in a multi-slab RW3 phantom, giving encouraging results, but, in principle, the situation in patient is far more complex. In addition, PRIMO was properly driven to reproduce an IMRT field, making use of in-house developed third party software, adapted to specific situations. The clinical implementation of IMRT simulation should be fast and easy to configure by the users. Consequently, the non-standard application, using external code as described in this work, is not the most suitable choice as it is, to help and assist the work of the Medical Physics Departments to perform MC simulations in clinical routine. The application being developed so far was used for research and development purposes and to study and adapt PRIMO to IMRT simulations. Further development of the configuration application should extend the application to other dynamic parameters, such as the gantry angle, in order to allow MC simulations of VMAT treatments. In addition, it is desirable to include tools for easy interaction between the user and the software, such as graphical user interfaces (GUI). Nevertheless, MC simulations are well known to be time consuming and this aspect still remains an open issue.

## Conclusions

The numerical model of the Varian Trilogy and Novalis equipment in the PRIMO software, including LINAC head and jaws/MLC collimation, are validated. Two algorithms were developed to drive PRIMO to divide a dynamic IMRT procedure into a definite number of static fields. The fixed step and random sampling algorithm were assessed on specific cases. A first basic IMRT test proved that PRIMO satisfactorily simulates a procedure with leaves moving with constant speed. The two algorithms are equivalent, under the condition of sufficiently high number of static fields. Both the algorithms in a more complex procedure showed matching results with respect to the experimental data and small differences between each other. When applied to an in phantom IMRT arbitrary field of a prostate treatment, the random sampling algorithm showed agreement with the experimental data. PRIMO was successfully driven to simulate an IMRT field, but the application developed so far requires adaptation for a user-friendly interaction between user and software.

The good results of the simulated dose distributions with respect to the experimental data depend on the specific arrangement. In addition, the calculation time resulted dependent by the number of static fields in use. The more the static fields to reproduce the dynamic MLC motion and the better the quality of the simulated dose distribution. Conversely, the post-processing time is not negligible with respect to the time for particle tracking simulation and interferes with the performances. The more static fields, the more time consuming the process is. A compromise in this work suggested using 50 static fields, but this result cannot be extended to general dynamic procedure, as it was verified in a specific IMRT field configuration.

## Abbreviations

3DCRT: 3 Dimensional Conformal Radiation Therapy
DICOM: Digital Imaging and Communications in Medicine
IMRT: Intensity Modulated Radiation Therapy
LINAC: Linear Accelerator
MC: Monte Carlo
MLC: Multi Leaf Collimator
MU: Monitor Unit
PDD: Percentage Depth Dose
phsp: phase space
PPS: Position-Probability Sampling
QA: Quality Assurance
RT: Radiation Therapy
SCS: Static Component Simulation
SSD: Source Surface Distance
TPS: Treatment Planning System
